# AGRASS Questionnaire: Assessment of Risk Management in Health Care

**DOI:** 10.11606/s1518-8787.2020054001335

**Published:** 2020-02-05

**Authors:** Zenewton André da Silva Gama, Pedro Jesus Saturno-Hernandez, Anna Claudia Sales Gomes Caldas, Marise Reis de Freitas, Ana Elza Oliveira de Mendonça, Carlos Alexandre de Souza Medeiros, Wilton Rodrigues Medeiros, Oliver Kessler, Diogo Penha Soares

**Affiliations:** I Universidade Federal do Rio Grande do Norte Centro de Ciências da Saúde Departamento de Saúde Coletiva NatalRN Brasil Universidade Federal do Rio Grande do Norte. Centro de Ciências da Saúde. Departamento de Saúde Coletiva. Natal, RN, Brasil; II National Institute of Public Health of Mexico CuernavacaMorelos Mexico National Institute of Public Health of Mexico. Cuernavaca, Morelos, Mexico; III Universidade Federal do Rio Grande do Norte Programa de Pós-Graduação em Saúde Coletiva NatalRN Brasil Universidade Federal do Rio Grande do Norte. Programa de Pós-Graduação em Saúde Coletiva - PPGSCOL. Natal, RN, Brasil; IV Universidade Federal do Rio Grande do Norte Centro de Ciências da Saúde Departamento de Infectologia NatalRN Brasil Universidade Federal do Rio Grande do Norte. Centro de Ciências da Saúde. Departamento de Infectologia Natal/RN, Brasil; V Universidade Federal do Rio Grande do Norte Centro de Ciências da Saúde Departamento de Enfermagem NatalRN Brasil Universidade Federal do Rio Grande do Norte. Centro de Ciências da Saúde. Departamento de Enfermagem. Natal, RN, Brasil; VI Empresa Brasileira de Serviços Hospitalares Hospital Universitário Onofre Lopes Unidade de Gestão de Riscos Assistenciais NatalRN Brasil Empresa Brasileira de Serviços Hospitalares. Hospital Universitário Onofre Lopes. Unidade de Gestão de Riscos Assistenciais Natal, RN, Brasil; VII Empresa Brasileira de Serviços Hospitalares Hospital Universitário Ana Bezerra Setor de vigilância em saúde e segurança do paciente Santa CruzRN Brasil Empresa Brasileira de Serviços Hospitalares. Hospital Universitário Ana Bezerra. Setor de vigilância em saúde e segurança do paciente. Santa Cruz, RN, Brasil; VIII Lucerne University of Applied Sciences and Arts Lucerne Switzerland Lucerne University of Applied Sciences and Arts. Lucerne, Switzerland; IX Agência Nacional de Vigilância Sanitária Gerência Geral de Tecnologia em Serviços de Saúde BrasíliaDF Brasil Agência Nacional de Vigilância Sanitária. Gerência Geral de Tecnologia em Serviços de Saúde. Brasília, DF, Brasil

**Keywords:** Patient Safety, Risk Management, Validation Studies

## Abstract

**OBJECTIVE:**

This study aims to assess the development and the validity analysis of the Assessment of Risk Management in Health Care Questionnaire (AGRASS).

**METHODS:**

This is a validation study of a measurement instrument following the stages: 1) Development of conceptual model and items; 2) Formal multidisciplinary assessment; 3) Nominal group for validity analysis with national specialists; 4) Development of software and national pilot study in 62 Brazilian hospitals 5) Delphi for validity analysis with the users of the questionnaire. In stages 3 and 5, the items were judged based on face validity, content validity, and utility and viability, by a 1-7 Likert scale (cut-off point: median < 6). Accuracy and reliability of the questionnaire were analyzed with the Confirmatory Factor Analysis and the Cronbach’s alpha.

**RESULTS:**

The initial version of the instrument (98 items) was adapted during stages 1 to 3 for the final version with 40 items, which were considered relevant, of adequate content, useful, and viable. The instrument has 2 dimensions and 9 subdimensions, and the items have closed-ended questions (yes or no). The software for the automatic collection and analysis generates indicators, tables, and automatic graphs for the assessed institution and aggregated data. The adjustment indices confirmed a bi-dimensional model composed of structure and process (X2/gl = 1.070, RMSEA ≤ 0.05 = 0.847, TLI = 0.972), with high reliability for the AGRASS Questionnaire (α = 0.94) and process dimension (α = 0.93), and adequate for the structural dimension (α = 0.70).

**CONCLUSIONS:**

The AGRASS Questionnaire is a potentially useful instrument for the surveillance and monitoring of the risk management and patient safety in health services.

## INTRODUCTION

The quality of health care is on the global health agenda, and patient safety is one of its critical components^1^. In order to improve the quality of health care by increasing the patient safety, health care systems are suggested to implement risk management practices^2^ and quality management/improvement methods applied to the health care safety^5^.

The risk management in health care is highlighted as one of the seven stages towards patient safety in the National Patient Safety Agency in the UK^2^. The National Quality Forum of the United States also includes identifying and reducing risks and hazards as the fourth of its 34 evidence-based safe practices. In the Brazilian health policy context, risk management in health facilities is the first objective of the *Programa Nacional de Segurança do Paciente* (PNSP – National Patient Safety Program)^4^.

The relationship between patient safety and quality of care occurs in two approaches: in the first one, patient safety is considered a dimension of the quality of health care^8 , 9^; while in the second one, safety is considered an aspect of health care regardless of their quality^10^. Overall, the World Health Organization^6^ (WHO) includes quality improvement methods as one of the 11 key topics of the multi-professional patient safety curriculum guide. In Brazil, the quality management focused on risk reduction is present in the regulation of health care^7 , 11^.

Considering the regulatory framework of the Brazilian health care, sanitary surveillance professionals are properly supported to oversee the implementation of quality management^11^ and risk management in health care^12^. The two management models can be used to improve safety. The external and internal sanitary inspectors and accountants need to have a wide and flexible insight to identify the implementation of principles and the achievement of objectives of the patient safety management, and not only a registry judgment of one model or another.

Many risk management and quality management models have been suggested for organizations in general^13^, developing many variations to health care. However, few studies devoted themselves to validating instruments to assess the implementation of quality^14^ and risks^15^management activities, showing the need of the area, especially in Brazil, which has no validated instrument for its local reality.

In a partnership between the *Agência Nacional de Vigilância Sanitária* (ANVISA – National Sanitary Surveillance Agency), the Pan American Health Organization (PAHO), and two universities, a combined model was suggested based on the principles of risk management and the quality of patient safety^14^. Based on this model, the Assessment of Risk Management in Health Care (AGRASS) was established, aiming to coordinate external sanitary audits and inspections performed in the Brazilian context and assisting the self-assessment of health care. Considering this background, this study aims to assess the development and the validity analysis of the AGRASS Questionnaire.

## METHOD

**Figure 1:**
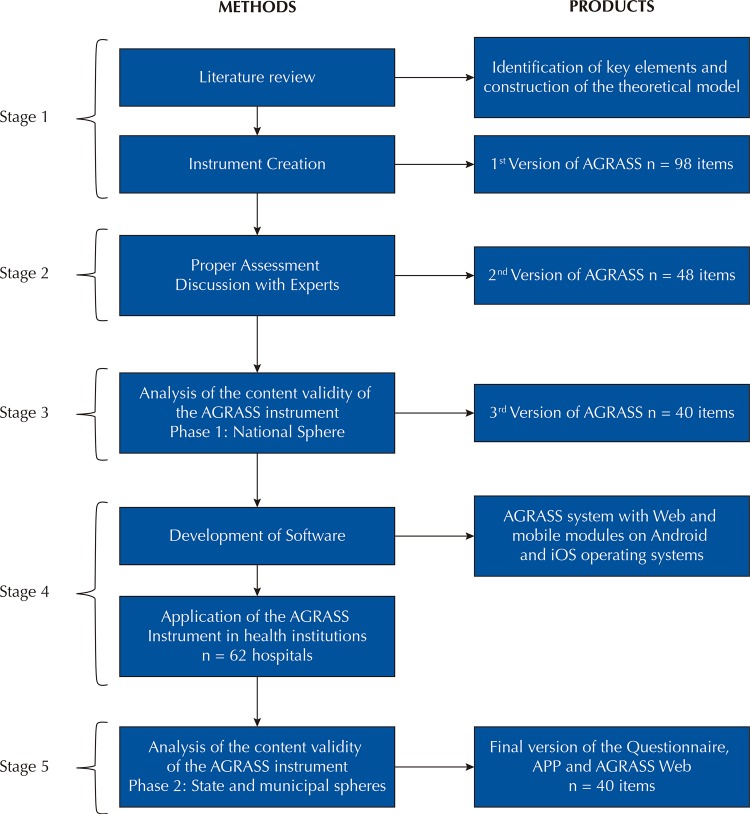
Stages of the construction and validation of the AGRASS Questionnaire..

Source: Gama ZAS, Saturno-Hernández PJ. Inspection of Risk Management Good Practices in Health Services. Natal: SEDIS-UFRN; 2017.

This is a validation study with a quality and quantity approach. The study was developed in partnership between ANVISA, PAHO, the Universidade Federal do Rio Grande do Norte (UFRN) in Brazil, and the Universidad de Murcia in Spain. This article describes the development and validation of the AGRASS Questionnaire in five stages, as shown in Figure 1 .

### Stage 1 – Development of the Conceptual Model and Items

An original and combined conceptual model was developed to show the key structures and processes of patient safety management in health care. This model was based on: wide insight of the literature on national and international technical documents; risk and quality management; models to improve the quality and risk management in health care^16^; *benchmarking* risk management publications in European health care^20^; international recommendations for patient safety practices that included risk management^2 , 3^; and Brazilian sanitary legislation^4 , 11 , 12^. This stage was performed in 2016 and had the initial dimension consensus by two doctoral researchers, who are specialists in management and improvement of quality in health care, and a third professional, who is a quality manager in a hospital certified by the National Accreditation Organization (NAO). The details of the reviewed references and of the conceptual model are available in a previous publication^13^.

### Stage 2 – Proper Assessment

The basic version of the AGRASS Questionnaire was properly reviewed by a group of six experts. The method of analysis was qualitative, by detailed discussion of the questionnaire items, aiming to adjust it to the best systems of patient safety management and considering the Brazilian context. The method should also be concise and consistent with the requirements of the sanitary surveillance. For the saturation analysis and consensus, three meetings were needed, developing the second version of the AGRASS Questionnaire in July 2017.

### Stage 3 – Validity Analysis with National Sphere Specialists

The second version was globally analyzed and reviewed item per item by ten ANVISA experts in August 2017. For the consensus, the nominal group technique^21^ was used, with three voting moments (two of them were face-to-face and the other one by distance). The questionnaire items were judged according to four criteria related to face validity, content validity, utility, and feasibility. The criteria were formulated in the form of closed-ended questions on a Likert scale from 1 to 7, on which 1 was “totally disagree” and 7 “totally agree”. The questions were the following: “Is the item relevant to the risk management in health care?,” “Is the item related to the dimension it wants to measure?,” “Is the item feasible in the context of risk management in health care?,” and “Is the feedback information useful to detect improvement opportunities in the risk management in care health?.” Each expert could also include comments regarding each item. This stage was useful for removing items, adapting terms, and adding fundamental item clarifications, developing the third version of the AGRASS Questionnaire.

### Stage 4 – Development of Software and National Pilot Study

With the product of the previous stage, the Laboratory of Technological Innovation in Health of UFRN developed the AGRASS System, which consists of two modules – mobile and web. The mobile module provides the questionnaire to be answered in health units, stores them, and sends the inserted responses to the web module. The web module automatically analyzes the data and provides individual or aggregate reports (sets of health care by region, state, municipality, and other groupings of institutions) with the report of the assessed health care and the indicators for the implementation of risk management, the latter showed in Table, Radar chart, and Pareto chart. The degree of risk management implementation in the health care assessed can be described according to individual items and group items. The items allow a positive response and are all equally weighted for the total estimate of risk management implementation in health care. For the descriptive analyses of group items, the percentage of positive responses is estimated, namely, the total positive responses in the group items regarding all items of the AGRASS Questionnaire or all items of the dimensions and subdimensions.

In possession of the electronic format of the AGRASS Questionnaire, a distance learning course was offered for 120 state and municipal professionals of the sanitary surveillance of all Brazilian federation, between September and December of 2017, based on the *ad hoc* didactic material that contains the AGRASS conceptual model. With the help and supervision of four researchers, the trained professionals applied the AGRASS Questionnaire in health care, which established the pilot study and the assessment of the offered course. The sample size was defined in a stratified manner and proportional to the amount of hospitals with intensive care unit (ICU) in each health care system of Brazil. The professionals made their choice conveniently seeking the largest services of their locality. Sixty-two hospitals participated in the pilot study. All of them had an ICU, because this type of service is considered a priority for sanitary surveillance, within the framework of the *Plano Integrado para a Gestão Sanitária da Segurança do Paciente* (Integrated Plan for Patient Safety Sanitary Management)^22^.

### Stage 5 – Validity Analysis with Instrument Users

Finishing the validation stages, the 74 professionals who applied the instrument were invited to participate in a validity analysis using the Delphi technique^21^. The examination was performed with an electronic validation form sent by e-mail, which included questions about the AGRASS Questionnaire using the same guidelines, criteria, and cut-off points of the validity analysis of stage 3. This examination occurred in February 2018, within 23 days, during which up to three reminders were sent to the non-respondents (10, 15, and 20 days after the examination).

### Data analysis

The validity analysis of the questionnaire items had quantitative and qualitative components. The data analysis in stages 3 and 5 considered the median of each of the four criteria. The items that obtained a median equal to or greater than 6 in all criteria were included in the final version of the AGRASS Questionnaire. The items that had some criterion with a median lower than 6 were discussed by the respective groups, and may have undergone adjustments, being submitted to a retrial by the experts. If the item had any medians lower than 6 in the second vote, it was removed. As for the qualitative component, the comments recorded in the assessment form and in the discussion after the presentation of the results of each vote were considered, even if the criterion had reached the cut-off point.

For the reliability analysis, internal consistency was estimated using the Cronbach’s alpha (Appendix 1) for the complete AGRASS Questionnaire. The structure and process dimensions were estimated using the questionnaire responses of the pilot study.

The validity and the bi-dimensional model of the questionnaire were assessed with Confirmatory Factor Analysis (CFA). The structural equation modeling was used to consider the adjustment of the observed data to the structure and process dimensions of risk management in health care. The robust estimation method was applied by Weighted Least Square Mean and Variance Adjusted (WLSMV) with the MPlus v.7 software (Muthén & Muthén). The measures used to verify the adequacy of the model to the data were: (i) Chi-square Ratio/Degrees of Freedom (χ^2^/gl), (ii) Root Mean Square Error of Approximation (RMSEA), (iii) Tucker-Lewis Index (TLI), (iv) Composite Reliability (CC), and (v) Average Variance Extracted (AVE). The reference values considered for a good adjustment were χ^2^/gl < 3.0; RMSEA < 0.05; TLI > 0.95; CC ≥ 70, and VME ≥ 50. For the RMSEA, in an ideal situation, the lower value of the 90% confidence of interval (90%CI) includes or approaches zero or is not greater than 0.05; and the higher value is not very large, namely, it is lower than 0.08^23^ (Appendix 2).

### Ethical aspects

This study was approved by the ethics committee in local research, with the opinion number 75662517.2.0000.5292, and it followed the requirements established for its realization.

## RESULTS

### Conceptual Model

**Figure 2:**
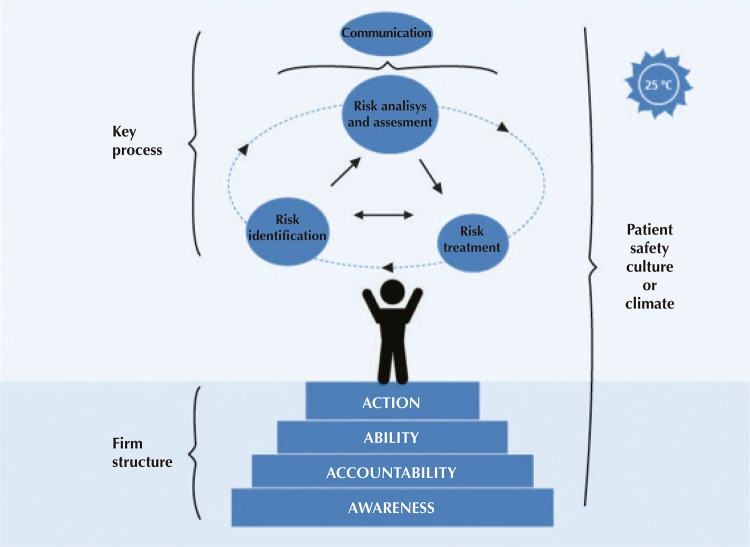
Conceptual model of the AGRASS Questionnaire.

The conceptual model developed in the first stage of the AGRASS Questionnaire regards the set of structures and processes that aim to constantly improve patient safety ( Figure 2 ). The dimension called “firm structure” consists of five subdimensions: awareness, accountability, ability, action, and safety culture. Additionally, the dimension called “key processes” consists of four subdimensions: risk identification, risk analysis and assessment, risk treatment , and risk communication. The processes joined by arrows represent the natural flow of integration between them.

The conceptual model aimed to present the practices of risk management in health care in a playful way and, for this, used an analogy with the art of a juggler ( Figure 2 ). In this figure, the juggler represents the person responsible for the patients’ safety in health care, as well as all other professionals involved. The balls balanced in the air represent the risk management processes, which must be fully present and integrated with each other. The firm soil and pleasant temperature, contextual factors that help the artist’s work, show the importance of the presence of leadership structures and systems and favorable organizational psychology regarding climate and safety culture.

### Proper Assessment and Validity Reviews

The first version of the AGRASS Questionnaire developed in stage 1 included 98 items regarding the dimensions of the conceptual model and was submitted to proper assessment in stage 2. After the analysis by six experts, 50 items were removed and a second version of the AGRASS Questionnaire with 48 items was developed.

The second version was submitted, in stage 3, to the validity analysis by 10 ANVISA professionals, whose aspects are shown in Table 1 . After the first voting round, 12 items did not reach the cut-off point and underwent discussion before a second vote. After the second voting round, only two items obtained a median lower than 6 and were therefore removed. Three other items were removed, even reaching medians greater than or equal to 6, because of the agreement among the experts’ group on whether they were disposable questions. Four items merged into a single item and 26 of them underwent adjustments. Chart 1 shows the results of this stage.

**Table 1:** Description of the participants of the AGRASS questionnaire validity analysis.

Variable	Stage 3 ANVISA n=10		Stage 5 State and municipal visas N=32	
	
	Number	Percentage	Number	Percentage
Sex				
Female	6	60%	30	93.7%
Male	4	40%	2	6.3%
Age				
< 30 years old	-	-	1	3.1%
31 to 40 years old	9	90%	11	34.4%
41 to 50 years old	1	10%	12	37.5%
51 to 60 years old	-	-	8	25.0%
Professional Training				
Architecture	-	-	1	3.1%
Biology	1	10%	-	-
Nursing	4	40%	22	68.8%
Engineering	-	-	1	3.1%
Pharmacy	1	10%	3	9.4%
Physics	1	10%	-	-
Physical therapy	-	-	2	6.2%
Medicine	-	-	2	6.2%
Dentistry	3	30%	-	-
Collective Health	-	-	1	3.1%
Graduate Degree				
None	1	10%	3	9.4%
Graduate Specialization	5	50%	19	59.4%
Masters	3	30%	8	25.0%
PhD	1	10%	2	6.2%
Service time				
< 5 years	3	30%	11	34.4%
6 to 10 years	3	30%	9	28.1%
11 to 15 years	4	40%	7	21.8%
> 15 years	-	-	5	15.7%
NSSS scope*				
National	10	100%	-	-
State	-	-	18	56.2%
Municipal	-	-	16	43.7%

ANVISA: National Sanitary Surveillance Agency

*NSSS: National Sanitary Surveillance System

**Chart 1:** Results of the validity analysis of the AGRASS Questionnaire and changes in terminology in the intermediate versions of the instrument.

VERSION 2	Median 1st Vote / 2nd Vote† n = 10				VERSION 3:	Median Single Vote† n = 32			
	
	C1	C2	C3	C4		C1	C2	C3	C4
**PART I – STRUCTURE FOR THE RISK MANAGEMENT ASSISTANCE**					**PART I – STRUCTURE FOR THE RISK MANAGEMENT ASSISTANCE**				
**Awareness**					**Awareness**				
1. Did the hospital promote any awareness-raising action for patient safety in the last year (event, campaign, etc.)?	7	7	7	6	1. Has the institution promoted any awareness-raising action for patient safety **in the last 12 months** (event, campaign, etc.)?	7	7	7	7
2. Are there posters, folders, posters or videos in the hospital drawing attention to patient safety?	5/7	7/7	7/7	5/6	2. Are there **in the institution** posters, folders, posters or videos drawing attention to patient safety?	7	7	7	7
**Accountability**					**Accountability**				
3. Is there an organizational unit responsible for improving patient safety (called from now on National Patient Safety Program – PNSP)?	7	7	7	7	3. Is there an organizational unit responsible for **coordinating the actions of** patient safety (called from now on to National Patient Safety Program – PNSP)?	7	7	7	7
4. Does it have a PNSP coordinator been appointed?	6	7	7	6,5	4. No adjustment	7	7	7	7
5. Does the PNSP have a record of at least six meetings in the last 12 months?	5/6	6/6	5/6	5/6	Eliminated after group discussion				
6. Is there a National Patient Safety Plan running?	7	6,5	7	7	5. No adjustment	7	7	7	7
**Ability**					**Ability**				
7. Does the NSP have professionals with formal workload dedication to risk management activities?	6,5	7	6,5	6	6. Does **the institution** have professionals with formal workload dedication to risk management activities?	7	7	7	7
8. Does the PNSP coordinator have exclusive dedication to risk management activities?	6/6	6/7	5/6	5/6	Eliminated after group discussion				
9. Is there allocation of financial resource for promotion of patient safety actions?	6/6	6/6	4/5	6/5	Eliminated				
10. Does the PNSP have adequate physical infrastructure?	5/6	5/6	4/6	4/7	7. No adjustment	7	7	7	7
11. Does the hospital provide adequate inputs for risk management actions?	4/6	6/6	5/7	5/7	8. Does **the institution ** provide adequate inputs for risk management actions?	7	7	7	7
12. Did the hospital promote training to its professionals in the area (risk management, quality management, patient safety, etc.)?	7	7	7	7	9. Did **the institution ** promote training to its professionals in the area (risk management, quality management, patient safety, etc.)?	7	7	7	7
**Safety Culture**					**Promoting patient safety culture**				
13. Was the safety culture assessed in the last 12 months?	5/7	7/7	5/7	6/7	10. Was the patient safety culture assessed in the last **24 months** ?	7	7	7	7
14. If it assessed the safety culture, were the results reported to clinical, administrative and care professionals?	5/7	7/7	7/7	6/7	11. **Did it communicate the results of the patient’s safety culture assessment to clinical, administrative and care professionals?**	7	7	7	7
15. If it assessed the safety culture, were implemented any interventions to improve the results identified in the assessment?	7	7	6,5	7	12. **Did it implement any intervention to improve the results identified in the assessment of the patient’s safety culture?**	7	7	7	7
**PART 2 – PROCESSES FOR THE RISK MANAGEMENT ASSISTANCE Risk identification**					**PART 2 – PROCESSES FOR THE RISK MANAGEMENT ASSISTANCE Risk identification**				
16. Does the hospital have a general list of the care risks identified in the institution?	6	7	7	7	Eliminated after group discussion				
17. Does it use an internal system to notify incidents?	6	7	6	7	13. No adjustment	7	7	7	7
18. Did the hospital disclose a list of sentinel events or never events for notification among professionals?	6	6,5	7	6	14. No adjustment	7	7	7	7
19. Does it monitor adherence to international patient safety goals?	6,5	6	6	6	15. Does it **monitor indicators of ** adherence to international patient safety goals?	7	7	7	7
20. Does the hospital use information on complaints and appeals (ombudsman’s office) to identify risks?	7	7	7	7	16. No adjustment	7	7	7	7
21. Does it use triggers or result tracker indicators to identify risks?	7/6	7/7	5/7	6/7	17. Does it use tracker indicators ( **clues to the existence of security incidents** ) or result indicators to identify risks?	7	7	7	7
22. Does it have a death review committee with meetings in the last six months (minutes)?	6	6	6	6	18. **Does it use the information on the death review committee to identify risks?**	7	7	7	7
23. Does it use the litigious processes of the hospital for risk identification?	6/7	6/7	6/7	5/7	19. Does it analyzes the litigious processes of the hospital for risk identification?	7	7	7	7
24. Does it use direct observation to identify risks? (e.g. examination of hand hygiene, contact precautions with patients in isolation, protective barriers in the insertion of Central Venous Catheter, etc.)?	7	6	7	7	20. No adjustment	7	7	7	7
25. Does it use electronic alert or support system for decision-making in electronic medical records (e.g. drug interactions, standardized discharge recommendations for specific patients, etc.)?	5/6	5/7	6/6	7/6	21. **Does it use electronic alert system in electronic medical records (e.g. drug interactions, standardized discharge recommendations for specific patients etc.)?**	7	7	7	7
26. Does it use checklists for patient safety?	7	7	7	7	22. No adjustment	7	7	7	7
27. Does it use risk mapping?	7	7	7	7	23. Did it **carry out** risk mapping of the health service?	7	7	7	7
28. Do security officials conduct security rounds in sectors to identify risks?	6	7	7	6	24. ** Are** patient safety rounds performed in the sectors to identify risks?	7	7	7	7
29. Does it confirm the use of external source to identify possible risks of at the institution (e.g. health alerts, media news, etc.)?	6	7	7	6	25. **Does it use external source for risk identification (e.g. health alerts, media news, etc.)?**	7	7	7	7
**Risk assessment analysis**					**Risk assessment analysis**				
30. Did it investigate (analysis of causes and contributing factors) any adverse events in the last 12 months?	7	7	7	7	26. **Did it perform analysis of causes and contributing factors for adverse events in the last 12 months?**	7	7	7	7
31. Does it use instruments for qualitative analysis of causes and contributing factors (flowchart, cause-effect diagram, force-field analysis, Bow Tie, brainstorming, etc.)?	7	7	7	7	27. No adjustment	7	7	7	7
32. Does it use instruments for quantitative analysis of causes or contributing risk factors (histogram, stratification, Pareto diagram and control chart)?	7	7	7	7	28. No adjustment	7	7	7	7
33. Does it use any risk prioritization matrix based on severity and frequency criteria?	7	7	7	7	29. No adjustment	7	7	7	7
34. Does it assess the adequacy of risk control or reduction measures?	7	7	7	7	Eliminated after group discussion				
**Risk treatment**					**Risk treatment**				
35. Did it implement basic clinical protocols for patient safety?	7	7	7	7	30. No adjustment	7	7	7	7
36. Did it implement action plans in reaction to investigated adverse events?	7	7	7	7	31. No adjustment	7	7	7	7
37. Does the hospital describe the responsible for the implementation for risk reduction actions?	7	7	7	7	**Questions adjusted in the 2nd vote to: 32. Does it present a complete action plan (schedule, responsible, resources and indicators) for risk reduction actions?**	7	7	7	7
38. Does the hospital describe the implementation schedule for risk reduction actions?	7	7	7	7					
39. Does the hospital describe and measure indicators of implementation and effectiveness of risk reduction actions?	6	7	7	7					
40. Does the hospital describe the resources needed for care risk reduction actions?	5/7	6/7	6/7	6/7					
**Risk communication**					**Risk communication**				
41. Does the high management receive periodic communication on the activities and results of care risk management?	7	7	7	7	**Do stakeholders of questions 33-35 receive periodic communication on the activities and results of care risk management?** 33. ** High Management** 34. ** Intermediary managers and clinical leaders** 35. ** Care professionals**	7	7	7	7
42. Do intermediary managers and clinical leaders receive periodic communication on the activities and results of care risk management?	7	7	7	7					
43. Do care professionals receive periodic communications on the activities and results of care risk management?	7	7	7	7					
44. Is the communication to patients about the adverse events (open disclosure of errors) that occurred standardized through any institutional norms, protocol or policy?	6	7	6	6	36. No adjustment	7	7	7	7
45. Does the hospital have sent external notifications (e.g. NOTIVISA) regularly in the last 12 months?	7	7	7	7	37. ** Does it perform external notification by the NOTIVISA system monthly?**	7	7	7	7
**Integration of risk management processes**					**Integration of risk management processes**				
46. Did the hospital perform a complete cycle of risk management (identification, analysis, assessment, treatment and monitoring of risk) in the last 12 months?	7	7	7	7	38. Did it perform a complete cycle of risk management (identification, analysis, assessment, treatment and monitoring) **or cycle of quality improvement focused on patient safety (PDCA, assessment and improvement cycle)** in the last 12 months?	7	7	7	7
47. Did it record the conduction of Root Cause Analyses or the London Protocol in the last 12 months?	6	7	6	7	39. Did the hospital conduct the Root Cause Analyses or the London Protocol in the last 12 months?	7	7	7	7
48. Did it record the conduction of Failure Mode and Effect Analysis (FMEA) in the last 12 months?	6	7	6	6	40. Did it **perform ** the Failure Mode and Effect Analysis (FMEA) in the last 12 months?	7	7	7	7

* C1 (Criterion 1) Is it relevant to the Assessment of Risk Management in Health Care (ARMHC)? C2 (Criterion 2) Does it relate to the subdimension you want to measure? C3 (Criterion 3) Is it feasible to assess ARMHC? C4 (Criterion 4) Is the information useful for detecting opportunities to improve the ARMHC?

† When no separation bars between the numbers are present, it means that only the first vote occurred.

The third version of the questionnaire had 40 items. An important contribution of this stage was the addition of the definition of each dimension and the descriptions about the items, to make the questionnaire clearer and more reliable. This version was used to develop the AGRASS System. Of the 120 sanitary surveillance technicians who enrolled in the course “Inspection of good risk management practices in health services,” 74 were approved and invited to participate in the second phase of the content validity analysis. Of these, 32 technicians signed an informed consent form and answered the validation study questionnaire. The participants’ description is shown in Table 1 . The group had members from all Brazilian regions (2 from the Midwest, 1 from the North, 7 from the Northeast, 17 from the Southeast, and 5 from the South). This group approved the relevance, usefulness, feasibility, and relationship with risk management of all 40 AGRASS Questionnaire items in the first vote, according to the results shown in Chart 1 .

The assessment measures of adjustments were used to verify the adequacy of the model to the data. It showed that the instrument is valid and appropriate to the two dimensions of structure and process, with < 3 chi-square ratio and degrees of freedom (X2/gl = 1.070). The RMSEA criterion reinforced the indication of good adjustment of the tested model (estimate = 0.037; 90%CI 0.000–0.057; probability of RMSEA ≤ 0.05 = 0.847). The TLI test (0.972), the composite reliability (0.931), and the extracted mean variance (0.563) also confirmed the structure of the two-factor model.

The standardized model shows that all items – except item 9, “The institution promoted its professionals training in the area (risk management, quality management, patient safety, etc.)” – presented significant factor loads (p < 0.05). However, as item 9 did not compromise the dimension model in general, it was kept in the questionnaire because of its attributed content validity by the experts.

Regarding the reliability analysis using Cronbach’s alpha, the AGRASS Questionnaire (40 items) had a result of 0.935 (high consistency), the structure dimension (12 items) had 0.704 (acceptable consistency), and the process dimension (28 items) had 0.931 (high consistency). The simulation of removal of items does not significantly change the internal consistency of the constructs assessed, showing that it is not necessary to remove or modify items from the final version.

### AGRASS Questionnaire – Final Version

The AGRASS Questionnaire has 40 items grouped into two dimensions and nine subdimensions of risk management in health care: structure (12 items grouped into four subdimensions) and processes (28 items grouped into five subdimensions), as shown in Table 2 . The AGRASS Survey (https://doi.org/10.6084/m9.figshare.7058141.v1), the AGRASS System (https://agrass.lais.huol.ufrn.br), which contains links to the mobile module in the Apple Store and Google Play, and an example of automatic report produced by the system (https://doi.org/10.6084/m9.figshare.7045454.v1) are available online.

**Table 2:** Dimensions and subdimensions of the AGRASS Questionnaire.

Dimension	Subdimension	Formulation of subdimension	No. of items
Structure (12 items)	Awareness	Professionals’ involvement in general, aiming the team’s commitment to the risk management in health care.	2
	Accountability	Definition of an organizational system responsible for the risk management assistance and accountability.	3
	Ability	Necessary resource provision to accomplish the risk management. Includes human resources in appropriate quantity and qualification, time for professionals to devote to activities of risk management, financial resources, equipment, and inputs.	4
	Promoting patient safety culture	Periodic assessment, feedback from employee assessments, and intervention in identified weaknesses, to constantly improve the patient safety culture.	3
Process (28 items)	Risk identification	Risk identification shows the manageable risks. Risk identification may be: retrospective , which identifies previous incidents in the institution and their probability of repetition; in real-time , which identifies risks at the critical time when a support incident may occur; and prospective , which identifies risks based on the future possibility of incidents, although they have not yet occurred at the institution.	13
	Risk assessment and analysis	Risk analysis and assessment activities aim to better understand safety problems before implementing risk reduction actions.	4
	Risk treatment	Planning activity and actions to improve patient safety, therefore avoiding, reducing, or transferring the risks.	3
	Risk communication	Constantly communicating the activities of risk management and the risks to managers, health professionals, patients, and external regulatory bodies.	5
	Process Integration	Risk management processes should be performed in a combined method, with or without the use of integration techniques (e.g. 5 whys, FMEA, etc.).	3
Total			40

5 whys: Five whys; FMEA: Failure Mode and Effect Analysis

## DISCUSSION

This study has a potential contribution to the improvement of surveillance and management of health care, as it provides a valid instrument for the assessment and monitoring of the organizational conditions that ensure the provision of health care insurance. After five stages of validation, the AGRASS Questionnaire was considered relevant, valid, useful, and feasible to measure the implementation of risk management in Brazilian health care. The questionnaire can be used to: assess health care regarding the implementation of risk management practices, guiding the planning of improvement interventions; review health care to measure improvement after performing interventions to implement risk management practices; and compare individual or aggregated health care (e.g. region, municipality, or state) regarding the level of risk management practices implementation. The instrument can be applied by external evaluators (sanitary inspectors, certifiers, accreditations and auditors, among others) or in self-assessment initiatives of health care systems.

### Contributions to Patient Safety

The AGRASS Questionnaire meets an international and national need. Information systems are one of the six main strategic interventions that WHO stimulates for the quality of health care^9^ and, particularly in the WHO Patient Safety Program, safety measurement is a priority objective^24^. However, even well-developed countries in patient safety, like England, have been urged to improve patient safety information collection^5^. This improvement opportunity was also evident in an assessment of government decisions regarding patient safety in countries of the Organization for Economic Co-operation and Development (OECD), which showed that assessment and monitoring are not very advanced^25^.

In Brazil, although the PNSP assessment and monitoring were planned^26^, there is a wide scope for improvement in this aspect. The *Sistema de Notificações para a Vigilância Sanitária* (NOTIVISA – Sanitary Surveillance Notification System) and the annual assessment of patient safety practices implemented by ANVISA are important, but insufficient^26 , 27^. Thus, the AGRASS Questionnaire presents itself as an alternative instrument to produce information for decision-making in patients’ safety based on 40 items, which can be comparable to 38 simple indicators and 2 composite indicators (items 15 and 30). This new instrument can be used to measure the patient safety at the level on which it has a greater lack of assessment, which is its organizational component. The quality of care should be monitored from a systemic perspective, including the technical level of individual care, but also the organizational levels of the establishment of health and the health care^18^. Patient safety indicators have been proposed for all levels^28 , 29^, but few are based on organizational criteria. The measurement of safety should be complete, not only based on adverse results or events^28 , 30^. Measuring the quality of care is not easy and requires balance.

### The Validity of the AGRASS Questionnaire

The stages of experts’ validation enabled us to reach the objectives of developing and validating the content of the AGRASS Questionnaire. In addition to the extensive review of the literature, the final items were approved regarding the face validity, which is the logical and clear importance for risk management; content validity, which is the ability of each item to measure the expressed concept of each dimension and subdimension of the questionnaire; and the construct validity, which measures how much the items adjust to the dimensional structure of the questionnaire. Content validation seeks to value opinion makers and recognizes the experts’ important contribution^23^. Regarding reliability, although it had acceptable internal consistency, it is emphasized that it depends on the evaluator’s training and on the correct application of the instrument, demanding and assessing the necessary vouchers. In addition, composite reliability values reinforce this aspect.

The name AGRASS Questionnaire emphasizes the risk management in health care, but it should be understood as an instrument for assessing patient safety management based on risk management and quality models. Regarding the size of the structure, this study highlights the structuring of leadership systems for patient safety and an environment defined by a favorable organizational culture. On the size of the risk management processes, those were included (identification, analysis and assessment, treatment, and communication) appear directly or indirectly in most risk and quality management models^13 , 16 , 28^. Risk identification subdimension includes retrospective methods (such as indicators), in real-time methods (such as checklists), and prospective methods (such as the safety walk-around)^3 , 13^. Another issue emphasized by the model is that processes should not be independent, but integrated with each other, showed in the subdimension “integration of processes.”

The fact that the AGRASS Questionnaire was originally developed for Brazilian health care favors its validity in the local context. Other instruments with similar objectives were developed abroad to evaluate the management of clinical risks^15^ and quality management^14^ but were not transculturally adapted to Brazil. Although these instruments seem similar to the AGRASS at first, they have important differences in their conceptual model and items.

### The AGRASS Electronic Assessment System

In order to help the periodic use of the AGRASS Questionnaire, the project sought to combine technological innovation to overcome usual barriers to the institutionalization of health care assessment processes in Brazil. As health care systems do not always have people with the time and skills to collect, analyze, and produce reports to guide decision-making, the AGRASS System helps these processes by providing a mobile application on Android and iOS systems. The application automatically provides service details and compliance tables regarding items, dimensions and subdimensions, as well as decision-making assessment charts. The web module provides the aggregation of individual assessments to measure the implementation in a network of services or geographic region. Reports are generated in .pdf files and databases in .xls files, remaining available for further analysis.

### Limitations and Future Studies

The AGRASS Questionnaire does not intend to thoroughly describe all safety management practices, but those most internationally recommended and nationally essential. Although it can be used to assist the inspection of sanitary standards, the instrument is not a complete inspection script, as it merely guides the assessment of risk management in health care.

It is suggested to conduct future descriptive studies of the application of this instrument in different regions of the national context, in addition to analyses of factors associated with good or poor results of implementation of risk management in health care.

## CONCLUSION

The AGRASS Questionnaire is an instrument considered valid after five stages of validation and potentially useful for the surveillance and measurement of the organizational structure for patient safety in Brazilian health care. The AGRASS System, composed of a mobile module and another web module, provides an efficient analysis of the implementation of risk management in health care, helping the institutionalization of the assessment focused on patient safety in health care and systems, which is a priority component and indispensable for the quality of health care.
