# Supplementary material for: AGRASS Questionnaire: Assessment of Risk Management in Health Care
Source: Rev Saude Publica. 2020 Feb 5;54:21. doi: 10.11606/s1518-8787.2020054001335 (PMC7006914; doi:10.11606/s1518-8787.2020054001335)
Supplement: Translation

# Translation: Questionário AGRASS: Avaliação da Gestão de Riscos Assistenciais em Serviços de Saúde

**Authors:** Zenewton André da Silva Gama, Pedro Jesus Saturno-Hernandez, Anna Claudia Sales Gomes Caldas, Marise Reis de Freitas, Ana Elza Oliveira de Mendonça, Carlos Alexandre de Souza Medeiros, Wilton Rodrigues Medeiros, Oliver Kessler, Diogo Penha Soares

**Affiliations:** I Brasil Universidade Federal do Rio Grande do Norte. Centro de Ciências da Saúde. Departamento de Saúde Coletiva. Natal, RN, Brasil; II Mexico National Institute of Public Health of Mexico. Cuernavaca, Morelos, Mexico; III Brasil Universidade Federal do Rio Grande do Norte. Programa de Pós-Graduação em Saúde Coletiva - PPGSCOL. Natal, RN, Brasil; IV Brasil Universidade Federal do Rio Grande do Norte. Centro de Ciências da Saúde. Departamento de Infectologia Natal/RN, Brasil; V Brasil Universidade Federal do Rio Grande do Norte. Centro de Ciências da Saúde. Departamento de Enfermagem. Natal, RN, Brasil; VI Brasil Empresa Brasileira de Serviços Hospitalares. Hospital Universitário Onofre Lopes. Unidade de Gestão de Riscos Assistenciais Natal, RN, Brasil; VII Brasil Empresa Brasileira de Serviços Hospitalares .Hospital Universitário Ana Bezerra. Setor de vigilância em saúde e segurança do paciente. Santa Cruz, RN, Brasil; VIII Switzerland Lucerne University of Applied Sciences and Arts. Lucerne, Switzerland.; IX Brasil Agência Nacional de Vigilância Sanitária. Gerência Geral de Tecnologia em Serviços de Saúde. Brasília, DF, Brasil

**Keywords:** Segurança do Paciente, Gestão de Riscos, Estudos de Validação

## Abstract

**OBJETIVO:**

O estudo objetiva descrever a construção e análise da validade do Questionário Avaliação da Gestão de Riscos Assistenciais em Serviços de Saúde (AGRASS).

**MÉTODOS:**

Trata-se de estudo de validação de um instrumento de medida nas etapas: 1. construção do modelo conceitual e itens; 2. apreciação formal multidisciplinar; 3. grupo nominal para análise da validade com especialistas da esfera nacional; 4. desenvolvimento de softwares e estudo-piloto nacional em 62 hospitais do Brasil; 5. Delphi para análise da validade com utilizadores do questionário. Nas etapas 3 e 5, os itens foram julgados quanto à validade de face e conteúdo, utilidade e viabilidade, em uma escala Likert de 1 a 7 (ponto de corte: mediana < 6). A validade de construto e a confiabilidade foram analisadas com análise fatorial confirmatória e coeficientes α de Cronbach.

**RESULTADOS:**

A versão inicial do instrumento (98 itens) foi adaptada durante as etapas 1 a 3 para a versão com 40 itens considerados relevantes, de conteúdo adequado, úteis e viáveis. O instrumento tem duas dimensões e nove subdimensões, e os itens têm opção de resposta fechada (sim ou não). Os *softwares* para coleta e análise automática geram indicadores, tabelas e gráficos automáticos para a instituição avaliada e conjuntos agregados. Os índices de ajuste confirmaram o modelo bidimensional de estrutura e processo (X2/gl = 1,070, RMSEA ≤ 0,05 = 0,847; TLI = 0,972), havendo confiabilidade alta para o Questionário AGRASS (α = 0,94) e a dimensão processo (α = 0,93) e aceitável para a dimensão estrutura (α = 0,70).

**CONCLUSÃO:**

O Questionário AGRASS é um instrumento potencialmente útil para a vigilância e monitoramento da gestão de riscos e segurança do paciente em serviços de saúde.

## INTRODUÇÃO

A qualidade do cuidado de saúde está na agenda de saúde global, e a segurança do paciente é um dos seus componentes críticos^1^. Visando melhorar a qualidade do cuidado de saúde pelo fortalecimento da segurança do paciente, tem sido recomendado que os sistemas e serviços de saúde implantem práticas de gestão de riscos^2^e métodos de gestão/melhoria da qualidade aplicados à segurança no cuidado de saúde^5^.

A gestão de riscos assistenciais em serviços de saúde é destacada como um dos sete passos para a segurança do paciente da *National Patient Safety Agency* do Reino Unido^2^. O *National Quality Forum* dos Estados Unidos também inclui a identificação e redução de riscos e perigos como a quarta de suas 34 práticas seguras baseadas em evidências. No âmbito da política de saúde brasileira, a gestão de riscos assistenciais em estabelecimentos de saúde é o primeiro dos objetivos específicos do Programa Nacional de Segurança do Paciente (PNSP)^4^.

A relação entre segurança do paciente e qualidade do cuidado se dá em duas abordagens: na primeira se considera a segurança do paciente uma dimensão da qualidade do cuidado de saúde^8 , 9^, enquanto na segunda a segurança é considerada um atributo dos serviços de saúde independente da qualidade^10^. Globalmente, a Organização Mundial da Saúde^6^(OMS) inclui os métodos de melhoria da qualidade como um dos 11 tópicos essenciais do guia curricular multiprofissional de ensino em segurança do paciente. No Brasil, o gerenciamento da qualidade com foco na redução de riscos está presente na regulamentação de serviços de saúde^7 , 11^.

Considerando o arcabouço regulatório dos serviços de saúde brasileiros, os profissionais da vigilância sanitária estão formalmente respaldados para fiscalizar a implantação do gerenciamento da qualidade^11^e do gerenciamento de riscos em serviços de saúde^12^. Os dois modelos de gestão podem ser utilizados para a melhoria da segurança, porém os inspetores sanitários e auditores externos e internos necessitam ter uma visão ampla e flexível que identifique a implantação dos princípios e alcance dos objetivos da gestão da segurança do paciente, não somente uma visão cartorial de processos de um modelo ou de outro.

Muitos modelos de gestão de riscos e de gestão da qualidade têm sido propostos para as organizações em geral^13^, gerando muitas adaptações aos serviços de saúde. No entanto, poucos estudos se dedicaram a validar instrumentos de avaliação da implantação de atividades da gestão da qualidade^14^e de riscos^15^, revelando uma necessidade da área, especialmente no Brasil, que não tem instrumento validado para a sua realidade local.

Em um trabalho de parceria entre a Agência Nacional de Vigilância Sanitária (Anvisa), a Organização Pan-Americana da Saúde (Opas) e duas universidades, foi proposto um modelo integrador baseado nos princípios de gestão de riscos e da qualidade para a segurança do paciente^14^. Baseado nesse modelo, construiu-se o Questionário Avaliação da Gestão de Riscos Assistenciais em Serviços de Saúde (AGRASS), com o objetivo de instrumentalizar auditorias e inspeções sanitárias externas realizadas no contexto brasileiro e auxiliar na autoavaliação de serviços de saúde. Considerando esses antecedentes, este estudo tem como objetivo descrever o processo de construção e análise da validade do Questionário AGRASS.

## MÉTODO

Este é um estudo de validação com abordagem quali-quantitativa. Foi desenvolvido em projetos de parceria entre a Anvisa, a Opas, a Universidade Federal do Rio Grande do Norte (UFRN) no Brasil e a Universidad de Murcia na Espanha. O presente artigo descreve o processo de construção e validação em cinco etapas, conforme apresentado na Figura 1 .

**Figura 1:** Etapas da construção e validação do Questionário AGRASS.

### Etapa 1 – Construção do Modelo Conceitual e dos Itens

Construímos um modelo conceitual original e integrador que representasse as estruturas e processos-chave da gestão da segurança do paciente em serviços de saúde. O modelo baseou-se em ampla revisão da literatura de documentos técnicos nacionais e internacionais sobre gestão de riscos e da qualidade, em modelos de melhoria da qualidade do cuidado de saúde e gestão de riscos em serviços de saúde^16^, em publicações de *benchmarking* de gestão de riscos em serviços de saúde europeus^20^, em recomendações internacionais de práticas de segurança do paciente que incluíam a gestão de riscos^2 , 3^e na legislação sanitária brasileira^4 , 11 , 12^. Essa etapa foi realizada em 2016, e o consenso inicial sobre as dimensões foi realizado por dois pesquisadores doutores, especialistas em gestão e melhoria da qualidade em serviços de saúde, e um terceiro profissional, gestor da qualidade em um hospital acreditado pela Organização Nacional de Acreditação. O detalhamento das referências revisadas e do modelo conceitual está disponível em uma publicação anterior^13^.

### Etapa 2 – Apreciação Formal

A versão inicial do Questionário AGRASS foi apreciada formalmente por um grupo de seis especialistas. O método de análise foi qualitativo, mediante discussão em profundidade sobre os itens do questionário, com o objetivo de ajustá-lo às melhores práticas de gestão da segurança do paciente e ser realista para o contexto brasileiro, condizente com as exigências da regulamentação sanitária e conciso. Para a saturação da análise e consenso, foram necessárias três reuniões em julho de 2017, gerando a segunda versão do Questionário AGRASS.

### Etapa 3 – Análise da Validade com Especialistas da Esfera Nacional

A segunda versão foi analisada de forma global e item a item por dez técnicos da ANVISA em agosto de 2017. Para o consenso, utilizou-se a técnica do grupo nominal^21^, com três momentos de votação, dois presenciais e um a distância. Os itens do questionário foram julgados segundo quatro critérios relacionados à validade de face, validade de conteúdo, utilidade e viabilidade. Os critérios foram formulados em forma de perguntas com opção de respostas fechadas em escala Likert de 1 a 7, sendo 1 “discordo totalmente” e 7 “concordo totalmente”. As perguntas foram as seguintes: “O item é relevante para a gestão de riscos assistenciais em serviços de saúde?”, “O item está relacionado com a dimensão que ele deseja medir?”, “O item é viável no contexto da gestão de riscos assistenciais em serviços de saúde?” e “A informação gerada pela resposta ao item é útil para detectar oportunidades de melhoria na gestão de riscos assistenciais em serviços de saúde?”. Cada especialista podia ainda incluir comentários a respeito de cada item. Essa etapa foi útil para eliminar itens, adaptar termos e acrescentar esclarecimentos fundamentais aos itens, gerando a terceira versão do Questionário AGRASS.

### Etapa 4 – Desenvolvimento dos Softwares e Estudo-Piloto Nacional

Com o produto da etapa anterior, o Laboratório de Inovação Tecnológica em Saúde da UFRN desenvolveu o Sistema AGRASS, que é composto por dois módulos. O módulo *mobile* provê o questionário a ser respondido nas unidades de saúde, armazena e envia as respostas inseridas para o módulo *web* , que analisa automaticamente os dados e disponibiliza relatórios individuais ou agregados (conjuntos de serviços de saúde por região, estado, município e outros agrupamentos de instituições) com as tabelas de caracterização do serviço de saúde avaliado e os indicadores de implantação da gestão de riscos, esses últimos representados em tabela, em gráficos de radar e em diagrama de Pareto. O grau de implantação da gestão de riscos assistenciais no serviço de saúde avaliado pode ser descrito segundo a conformidade dos itens individuais e por grupos de itens. Os itens possibilitam uma resposta positiva e todos têm o mesmo peso no cálculo da estimativa total de implantação da gestão de riscos assistenciais. Para análises descritivas de grupos de itens, calcula-se a porcentagem de respostas positivas, ou seja, total de respostas positivas no grupo de itens em relação ao total de itens do Questionário AGRASS ou ao total de itens das dimensões e subdimensões.

De posse do Questionário AGRASS em formato eletrônico, ofertou-se um curso a distância para 120 profissionais das vigilâncias sanitárias estaduais e municipais de todas as unidades da federação brasileira, entre setembro e dezembro de 2017, com base no material didático *ad hoc* que contém o modelo conceitual do AGRASS. Após o treinamento, com o auxílio e supervisão de quatro pesquisadores, os profissionais aplicaram o Questionário AGRASS em serviços de saúde, o que constituiu o estudo-piloto desse instrumento e também a avaliação do curso. O tamanho da amostra foi definido de forma estratificada e proporcional à quantidade de hospitais com unidade de terapia intensiva (UTI) em cada unidade da federação brasileira, e os profissionais fizeram a escolha por conveniência buscando os maiores serviços de sua localidade. Participaram do piloto 62 hospitais. Todos dispunham de UTI, pois este tipo de serviço é considerado prioritário para a vigilância sanitária, no âmbito do Plano Integrado para a Gestão Sanitária da Segurança do Paciente^22^.

### Etapa 5 – Análise da Validade com Utilizadores do Instrumento

Finalizando as etapas de validação, os 74 profissionais que aplicaram o instrumento foram convidados a participar de uma análise da validade utilizando a técnica Delphi^21^. A consulta foi realizada por meio de um formulário eletrônico de validação enviado por e-mail, que continha questões sobre o Questionário AGRASS utilizando as mesmas orientações, critérios e pontos de corte da análise da validade da etapa 3. Essa consulta ocorreu em fevereiro de 2018 por um prazo total de 23 dias, durante o qual foram enviados até três lembretes aos não respondentes (10, 15 e 20 dias depois da consulta).

### Análise dos Dados

A análise da validade dos itens do questionário teve um componente quantitativo e outro qualitativo. A análise dos dados nas etapas 3 e 5 considerou a mediana de cada um dos quatros critérios. Os itens que obtiveram mediana igual ou maior que 6 em todos os critérios foram incluídos na versão final do Questionário AGRASS. Os itens que tiveram algum critério com mediana inferior a 6 foram discutidos pelos respectivos grupos, podendo ter sofrido alteração em sua redação, e foram submetidos a novo julgamento pelos especialistas. Se o item teve alguma mediana inferior a 6 na segunda votação, ele foi eliminado. Quanto ao componente qualitativo, consideraram-se os comentários registrados no formulário de avaliação e na discussão após apresentação dos resultados de cada votação, mesmo que o critério tivesse alcançado o ponto de corte.

Para análise da confiabilidade, calculou-se a consistência interna mediante os coeficientes α de Cronbach (Apêndice 1) para o Questionário AGRASS completo e as dimensões de estrutura e de processo, utilizando as respostas dos questionários no estudo-piloto.

A validade de construto e o respectivo modelo bidimensional do questionário foram avaliados com análise fatorial confirmatória. Utilizou-se modelagem por equações estruturais para investigar o ajuste dos dados observados às dimensões de estrutura e processo da gestão de riscos assistenciais. Aplicou-se o método de estimação robusta por mínimos quadrados ponderados ajustados pela média e variância (WLSMV) com o software MPlus v.7 (Muthén & Muthén). As medidas utilizadas para verificar a adequação do modelo aos dados foram: (i) razão qui-quadrado/graus de liberdade (X^2^/gl), (ii) raiz do erro quadrático médio de aproximação (RMSEA), (iii) índice de ajuste de Tucker-Lewis (TLI), (iv) confiabilidade composta (CC) e (v) variância média extraída (VME). Os valores de referência considerados para um bom ajuste foram X^2^/gl < 3,0; RMSEA < 0,05; TLI > 0,95; CC ≥ 70 e VME ≥ 50. Para a RMSEA, em uma situação ideal, o valor inferior do intervalo de confiança de 90% (IC90%) inclui ou se aproxima muito de zero, ou não é maior que 0,05; e o valor superior não é muito grande, ou seja, apresenta-se menor que 0,08^23^(Apêndice 2).

### Aspectos Éticos

O estudo foi aprovado pelo comitê de ética em pesquisa local, com número de parecer 75662517.2.0000.5292, e seguiu os requisitos estabelecidos para sua realização.

## RESULTADOS

### Modelo Conceitual

O modelo conceitual produzido na primeira etapa de construção do Questionário AGRASS faz referência ao conjunto de estruturas e processos que objetivam melhorar continuamente a segurança do paciente ( Figura 2 ). A dimensão denominada “estrutura firme” é composta por cinco subdimensões: sensibilização, responsabilização, habilitação, ação e cultura de segurança. Adicionalmente, a dimensão denominada “processos chaves” é composta por quatro subdimensões: identificação de riscos, análise e avaliação de riscos, tratamento de riscos e comunicação de riscos. Os processos unidos por setas representam o fluxo natural de integração entre eles.

**Figura 2:** Modelo conceitual do Questionário AGRASS.

Fonte: Gama ZAS, Saturno-Hernández PJ. Inspeção de Boas Práticas de Gestão de Riscos em Serviços de Saúde. Natal: SEDIS-UFRN; 2017.

O modelo conceitual teve por objetivo apresentar as práticas de gestão de riscos assistenciais de forma lúdica e, para isso, utilizou uma analogia com a arte de um malabarista ( Figura 2 ). Nessa ilustração, o malabarista representa o responsável (ou responsáveis) pela segurança do paciente no serviço de saúde, assim como todos os outros profissionais envolvidos. As bolas equilibradas no ar representam os processos de gestão de riscos, que devem estar integralmente presentes e integrados entre si. O solo firme e a temperatura agradável, fatores contextuais que facilitam o trabalho do artista, ilustram a importância da presença de estruturas e sistemas de liderança e de uma psicologia organizacional favorável quanto ao clima e cultura de segurança.

### Apreciação Formal e Análises da Validade

A primeira versão do Questionário AGRASS produzida na etapa 1 continha 98 itens relativos às dimensões do modelo conceitual e foi submetida à apreciação formal na etapa 2. Após análise de seis especialistas, excluíram-se 50 itens e foi produzida uma segunda versão do Questionário AGRASS com 48 itens.

A segunda versão foi submetida à análise da validade na etapa 3 por 10 profissionais da Anvisa, cujas características estão presentes na Tabela 1 . Após a primeira rodada de votação, 12 itens não alcançaram o ponto de corte e passaram por discussão antes de uma segunda votação. Após a segunda rodada de votação, apenas dois itens obtiveram mediana inferior a seis e por isso foram retirados. Outros três itens foram retirados, mesmo alcançando medianas maiores ou iguais a 6, por consenso entre o grupo de especialistas sobre serem questões dispensáveis, enquanto quatro itens fundiram-se em um único item e 26 itens sofreram adaptações de redação. O Quadro 1 sintetiza os resultados dessa etapa.

**Tabela 1:** Caracterização dos participantes da análise da validade do Questionário AGRASS.

Variável	Etapa 3 Anvisa n=10		Etapa 5 Visas estaduais e municipais N=32	
	
	Número	Porcentagem	Número	Porcentagem
Sexo				
Feminino	6	60%	30	93,7%
Masculino	4	40%	2	6,3%
Idade				
< 30 anos	-	-	1	3,1%
31 a 40 anos	9	90%	11	34,4%
41 a 50 anos	1	10%	12	37,5%
51 a 60 anos	-	-	8	25,0%
Formação profissional				
Arquitetura	-	-	1	3,1%
Biologia	1	10%	-	-
Enfermagem	4	40%	22	68,8%
Engenharia	-	-	1	3,1%
Farmácia	1	10%	3	9,4%
Física	1	10%	-	-
Fisioterapia	-	-	2	6,2%
Medicina	-	-	2	6,2%
Odontologia	3	30%	-	-
Saúde Coletiva	-	-	1	3,1%
Pós-Graduação				
Nenhuma	1	10%	3	9,4%
Especialização	5	50%	19	59,4%
Mestrado	3	30%	8	25,0%
Doutorado	1	10%	2	6,2%
Tempo de serviço				
< 5 anos	3	30%	11	34,4%
6 a 10 anos	3	30%	9	28,1%
11 a 15 anos	4	40%	7	21,8%
> 15 anos	-	-	5	15,7%
Esfera do SNVS				
Nacional	10	100%	-	-
Estadual	-	-	18	56,2%
Municipal	-	-	16	43,7%

Anvisa: Agencia Nacional de Vigilância Sanitária; Visas: Vigilâncias Sanitárias

**Quadro1:** Resultados da análise da validade do Questionário AGRASS e mudanças de terminologia nas versões intermediárias do instrumento.

VERSÃO 2	Mediana 1º Voto / 2º Voto^†^n=10				VERSÃO 3	Mediana Único Voto^†^n=32			
	
	C1	C2	C3	C4		C1	C2	C3	C4
PARTE I - ESTRUTURA PARA A GESTÃO DE RISCOS ASSISTENCIAIS					PARTE I - ESTRUTURA PARA A GESTÃO DE RISCOS ASSISTENCIAIS				
Sensibilização					Sensibilização				
1. O hospital promoveu alguma ação de sensibilização para a segurança do paciente no último ano (evento, campanha, etc.)?	7	7	7	6	1. A instituição promoveu alguma ação de sensibilização para a segurança do paciente nos últimos 12 meses (evento, campanha, etc.)?	7	7	7	7
2. Existem no hospital cartazes, folders, pôsteres ou vídeos chamando atenção para a segurança do paciente?	5/7	7/7	7/7	5/6	2. Existem na instituição cartazes, folders, pôsteres ou vídeos chamando atenção para a segurança do paciente?	7	7	7	7
Responsabilização					Responsabilização				
3. Existe uma unidade organizacional responsável por melhorar a segurança do paciente (chamada a partir de agora de Núcleo de Segurança do Paciente - NSP)?	7	7	7	7	3. Existe uma unidade organizacional responsável por coordenar as ações de segurança do paciente (chamada a partir de agora de Núcleo de Segurança do Paciente - NSP)?	7	7	7	7
4. Foi nomeado um coordenador do NSP?	6	7	7	6,5	4. Sem alteração	7	7	7	7
5. O NSP tem registro de pelo menos seis reuniões nos últimos 12 meses?	5/6	6/6	5/6	5/6	Eliminada após discussão do grupo				
6.Existe um Plano de Segurança do Paciente em execução?	7	6,5	7	7	5. Sem alteração	7	7	7	7
Habilitação					Habilitação				
7. O NSP dispõe de profissionais com dedicação de carga horária formal às atividades de gestão de riscos?	6,5	7	6,5	6	6. A instituição dispõe de profissionais com dedicação de carga horária formal às atividades de gestão de riscos?	7	7	7	7
8. O coordenador do NSP tem dedicação exclusiva às atividades de gestão de riscos?	6/6	6/7	5/6	5/6	Eliminada após discussão do grupo				
9. Existe alocação de recurso financeiro para as ações de promoção da segurança do paciente?	6/6	6/6	4/5	6/5	Eliminada				
10. O NSP dispõe de uma infraestrutura física adequada?	5/6	5/6	4/6	4/7	7. Sem alteração	7	7	7	7
11. O hospital provê insumos adequados para as ações de gestão de riscos?	4/6	6/6	5/7	5/7	8. A instituição provê insumos adequados para as ações de gestão de riscos?	7	7	7	7
12. O hospital promoveu aos seus profissionais capacitação na área (gestão de riscos, gestão da qualidade, segurança do paciente etc.)?	7	7	7	7	9. A instituição promoveu aos seus profissionais capacitação na área (gestão de riscos, gestão da qualidade, segurança do paciente etc.)?	7	7	7	7
Cultura de Segurança					Promoção de Cultura de Segurança				
13. Avaliou a cultura de segurança nos últimos 12 meses?	5/7	7/7	5/7	6/7	10. Avaliou a cultura de segurança do paciente nos últimos 24 meses?	7	7	7	7
14. Se avaliou a cultura de segurança, comunicou os resultados às lideranças clínicas, administrativas e os profissionais da assistência?	5/7	7/7	7/7	6/7	11. Comunicou os resultados da avaliação da cultura de segurança do paciente às lideranças clínicas, administrativas e os profissionais da assistência?	7	7	7	7
15. Se avaliou a cultura de segurança, implantou alguma intervenção para melhorar os resultados identificados na avaliação?	7	7	6,5	7	12. Implantou alguma intervenção para melhorar os resultados identificados na avaliação da cultura de segurança do paciente?	7	7	7	7
PARTE II – PROCESSOS DE GESTÃO DE RISCOS ASSISTENCIAIS Identificação de riscos					PARTE II – PROCESSOS DE GESTÃO DE RISCOS ASSISTENCIAIS Identificação de riscos				
16. O hospital apresenta uma lista geral dos riscos assistenciais identificados na instituição?	6	7	7	7	Eliminada após discussão do grupo				
17. Utiliza um sistema interno de notificação de incidentes?	6	7	6	7	13. Sem alteração	7	7	7	7
18. Divulgou entre os profissionais uma lista de eventos sentinela ou never events para notificação?	6	6,5	7	6	14. Sem alteração	7	7	7	7
19. Realiza monitoramento da adesão às metas internacionais de segurança do paciente?	6,5	6	6	6	15. Monitora indicadores de adesão às metas internacionais de segurança do paciente?	7	7	7	7
20. Utiliza informações das queixas e reclamações (ouvidoria) para identificação de riscos?	7	7	7	7	16. Sem alteração	7	7	7	7
21. Utiliza indicadores rastreadores (tipo triggers) ou de resultado para identificar riscos?	7/6	7/7	5/7	6/7	17. Utiliza indicadores rastreadores (pistas da existência de incidentes de segurança) ou de resultado para identificar riscos?	7	7	7	7
22. Possui comissão de revisão de óbito com reuniões nos últimos seis meses (ata)?	6	6	6	6	18. Utiliza as informações da comissão de revisão de óbito para identificação de riscos?	7	7	7	7
23. Utiliza os processos litigiosos do hospital para identificação de risco?	6/7	6/7	6/7	5/7	19. Analisa os processos litigiosos da instituição para identificação de risco?	7	7	7	7
24. Utiliza observação direta para identificar riscos? (ex. observação da higiene das mãos, de precauções de contato com pacientes em isolamento, de barreiras de proteção na inserção de CVC, etc.)?	7	6	7	7	20. Sem alteração	7	7	7	7
25. Utiliza sistema eletrônico de alerta ou apoio à tomada de decisão em prontuário eletrônico (ex. interações medicamentosas, recomendações padronizadas de alta para pacientes específicos, etc.)?	5/6	5/7	6/6	7/6	21 Utiliza sistema eletrônico de alerta em prontuário eletrônico (ex. interações medicamentosas, recomendações padronizadas de alta para pacientes específicos, etc.)?	7	7	7	7
26. Utiliza checklists ou listas de verificação para a segurança do paciente?	7	7	7	7	22. Sem alteração	7	7	7	7
27. Utiliza o mapeamento de riscos?	7	7	7	7	23 Realizou mapeamento de riscos do serviço de saúde?	7	7	7	7
28. Os responsáveis pela segurança realizam rondas de segurança nos setores para identificar riscos?	6	7	7	6	24 Realizam-se rondas de segurança do paciente nos setores para identificar riscos?	7	7	7	7
29. Comprova a utilização de fonte externa para a identificação de riscos possíveis de ocorrer na instituição (ex. alertas sanitários, notícias na mídia, etc.)?	6	7	7	6	25.Utiliza fonte externa para a identificação de riscos (ex. alertas sanitários, notícias na mídia, etc.)?	7	7	7	7
Análise de Avaliação de risco					Análise de Avaliação de risco				
30. Investigou (análise de causas e fatores contribuintes) algum evento adverso nos últimos 12 meses?	7	7	7	7	26. Realizou análise de fatores contribuintes para algum evento adverso nos últimos 12 meses?	7	7	7	7
31. Utiliza ferramentas de análise qualitativa das causas e fatores contribuintes (fluxograma, diagrama de causa-efeito, análise de campo de força, Bow Tie, brainstorming, etc.)?	7	7	7	7	27. Sem alteração	7	7	7	7
32. Utiliza ferramentas de análise quantitativa das causas ou fatores contribuintes dos riscos (histograma, estratificação, diagrama de Pareto e gráfico de controle)?	7	7	7	7	28. Sem alteração	7	7	7	7
33.Utiliza alguma matriz de priorização de riscos baseada em critérios de gravidade e frequência?	7	7	7	7	29. Sem alteração	7	7	7	7
34. Avalia a adequação das medidas de controle ou redução dos riscos?	7	7	7	7	Eliminada após discussão do grupo				
Tratamento de Risco					Tratamento de Risco				
35. Implantou os protocolos clínicos básicos para a segurança do paciente?	7	7	7	7	30. Sem alteração	7	7	7	7
36. Implementou planos de ação em reação a eventos adversos investigados?	7	7	7	7	31. Sem alteração	7	7	7	7
37. O hospital descreve os responsáveis pela implementação das ações de redução de risco?	7	7	7	7	Alteradas na 2ª votação para: 32. Apresenta plano de ação completo (cronograma, responsáveis, recursos e indicadores) para as ações de redução de riscos?	7	7	7	7
38. O hospital descreve o cronograma de implementação das ações de redução de risco?	7	7	7	7					
39. O hospital descreve e mede indicadores de implementação e efetividade das ações de redução de risco?	6	7	7	7					
40. O hospital descreve os recursos necessários às ações de redução de riscos assistenciais?	5/7	6/7	6/7	6/7					
Comunicação de riscos					Comunicação de riscos				
41. A alta gestão recebe comunicação periódica sobre as atividades e resultados da gestão dos riscos assistenciais?	7	7	7	7	Os interessados das questões 33-35 recebem comunicação periódica sobre as atividades e resultados da gestão dos riscos assistenciais? 33. Alta Gestão 34. Os gestores intermediários e lideranças clínicas 35. Os profissionais da assistência	7	7	7	7
42. Os gestores intermediários e lideranças clínicas recebem comunicação periódica sobre as atividades e resultados da gestão dos riscos assistenciais?	7	7	7	7					
43. Os profissionais da assistência recebem comunicações periódicas sobre as atividades e resultados da gestão dos riscos assistenciais?	7	7	7	7					
44. A comunicação aos pacientes sobre os eventos adversos (revelação aberta de erros) ocorridos está padronizada mediante alguma norma, protocolo ou política institucional?	6	7	6	6	36. Sem alteração	7	7	7	7
45. Enviou notificações externas (ex. NOTIVISA) regularmente nos últimos 12 meses?	7	7	7	7	38. Realiza notificação externa pelo sistema NOTIVISA mensalmente?	7	7	7	7
Integração dos processos de gestão de riscos					Integração dos processos de gestão de riscos				
46. Realizou um ciclo completo de gestão de riscos (identificação, análise, avaliação, tratamento e monitoramento do risco) nos últimos 12 meses?	7	7	7	7	38.Realizou ciclo completo de gestão de riscos (identificação, análise, avaliação, tratamento e monitoramento) ou ciclo de melhoria da qualidade focado na segurança do paciente (PDCA, ciclo de avaliação e melhoria) nos últimos 12 meses?	7	7	7	7
47. Documentou a realização de Análise de Causas-Raiz (ACR) ou Protocolo de Londres nos últimos 12 meses?	6	7	6	7	39. Realizou Análise de Causas-Raiz (ACR) ou Protocolo de Londres nos últimos 12 meses?	7	7	7	7
48. Foi documentada a realização de Análise dos Modos de Falhas e Efeitos (AMFE) nos últimos 12 meses?	6	7	6	6	40. Realizou Análise dos Modos de Falhas e Efeitos (AMFE ou *FMEA* ) nos últimos 12 meses?	7	7	7	7

* C1 (Critério 1): É relevante para a Gestão de Riscos Assistenciais em Serviços de Saúde (GRASS)? C2 (Critério 2): Relaciona-se com a subdimensão que deseja medir? C3 (Critério 3): É viável para avaliar a GRASS? C4 (Critério 4): A informação é útil para detectar oportunidades de melhoria na GRASS?

† Quando não houver barras de separação entre os números, significa que houve apenas a primeira votação.

A terceira versão do questionário ficou com 40 itens. Uma importante contribuição desta etapa foi o acréscimo da definição de cada dimensão e esclarecimentos a respeito dos itens, na tentativa de tornar o questionário mais claro e confiável. Essa versão foi a utilizada para construir o Sistema AGRASS. Dos 120 técnicos da vigilância sanitária que se inscreveram no curso “Inspeção de boas práticas de gestão de riscos em serviços de saúde”, 74 foram aprovados e convidados a participar da segunda fase da análise de validade de conteúdo. Desses, 32 assinaram o termo de consentimento e responderam ao questionário de estudo de validação. A caracterização dos participantes está descrita na Tabela 1 . O grupo teve representantes de todas as regiões do Brasil (2 do Centro-Oeste, 1 do Norte, 7 do Nordeste, 17 do Sudeste e 5 do Sul). Esse grupo aprovou a relevância, utilidade, viabilidade e relação com a gestão de riscos de todos os 40 itens do Questionário AGRASS na primeira votação, conforme resultados detalhados no Quadro 1 .

As medidas de avaliação dos ajustes usadas para verificar a adequação do modelo aos dados mostraram que o instrumento é válido e adequado às duas dimensões de estrutura e processo, com razão de qui-quadrado e graus de liberdade < 3 (X2/gl = 1,070). O critério RMSEA reforçou a indicação de bom ajuste do modelo testado (estimativa = 0,037; IC90% 0,000–0,057; probabilidade de RMSEA ≤ 0,05 = 0,847). O teste TLI (0,972), a confiabilidade composta (0,931) e a variância média extraída (0,563) também confirmam a estrutura do modelo de dois fatores.

O modelo padronizado mostra que todos os itens – exceto o item 9, “A instituição promoveu aos seus profissionais capacitação na área (gestão de riscos, gestão da qualidade, segurança do paciente, etc.)” – apresentaram cargas fatoriais significativas (p < 0,05). Entretanto, como o item 9 não comprometeu o modelo da dimensão em geral, foi mantido no questionário em virtude da validade de conteúdo atribuída pelos especialistas.

Quanto à análise da confiabilidade mediante os coeficientes α de Cronbach, o Questionário AGRASS (40 itens) teve resultado 0,935 (alta consistência), a dimensão estrutura (12 itens) teve 0,704 (consistência aceitável) e a dimensão processo (28 itens) teve 0,931 (alta consistência). A simulação da exclusão de itens não altera significativamente a consistência interna dos construtos avaliados, demonstrando que não é necessário retirar ou modificar itens da versão final.

### Questionário AGRASS – Versão Final

O Questionário AGRASS possui 40 itens agrupados em duas dimensões e nove subdimensões da gestão de riscos assistenciais: estrutura (12 itens agrupados em quatro subdimensões) e processos (28 itens agrupados em cinco subdimensões), conforme apresentado na Tabela 2 . O Questionário AGRASS (https://doi.org/10.6084/m9.figshare.7058141.v1), o Sistema AGRASS (https://agrass.lais.huol.ufrn.br), que contém os links para o módulo mobile na *Apple Store* e *Google Play* , e um exemplo de relatório automático gerado pelo sistema (https://doi.org/10.6084/m9.figshare.7045454.v1) estão disponíveis on-line.

**Tabela 2:** Dimensões e subdimensões do Questionário AGRASS.

Dimensão	Subdimensão	Definição da subdimensão	Nº itens
Estrutura (12 itens)	Sensibilização	Envolvimento dos profissionais em geral, para que a organização esteja comprometida com a gestão dos riscos assistenciais.	2
	Responsabilização	Definição de uma estrutura organizacional responsável pela gestão dos riscos assistenciais e prestação de contas de suas atividades.	3
	Habilitação	Provimento de recursos necessários para cumprir com a responsabilidade de gerenciar riscos. Inclui recursos humanos em quantidade e qualificação apropriada, tempo para os profissionais dedicarem às atividades de gerenciar riscos, recursos financeiros, equipamentos e insumos.	4
	Promoção da cultura de segurança	Avaliação periódica, feedback das avaliações aos colaboradores e intervenção nas fragilidades identificadas, para melhorar continuamente a cultura de segurança	3
Processo (28 itens)	Identificação de riscos	A identificação de riscos revela os riscos que podem ser gerenciados. Pode ser retrospectiva , que identifica incidentes prévios na instituição e sua probabilidade de recorrência; em tempo-real , que identifica riscos no momento crítico em que pode ocorrer incidente na assistência; e prospectiva , que identifica riscos com base na possibilidade futura de incidentes, apesar de que ainda não ocorreram na instituição.	13
	Análise e avaliação de riscos	Atividades de análise e avaliação dos riscos objetivam conhecer melhor os problemas de segurança antes de implementar ações de redução do risco.	4
	Tratamento de riscos	Atividade de planejamento e implementação de ações de melhoria da segurança, podendo evitar, reduzir ou transferir os riscos.	3
	Comunicação de riscos	Atividade de comunicar continuamente os riscos e atividades da gestão de riscos aos gerentes, profissionais de saúde, pacientes e órgãos reguladores externos.	5
	Integração dos processos	Os processos de gestão de riscos devem ser realizados de forma integrada, com ou sem uso de técnicas integradoras (ex. ACR, AMFE, etc.).	3
Total			40

ACR: Análise de Causa Raiz; AMFE: Análise dos Modos de Falhas e Efeitos

## DISCUSSÃO

Este trabalho tem um potencial de contribuição para a melhoria da vigilância e gestão de sistemas e serviços de saúde, pois disponibiliza um instrumento válido para a avaliação e monitoramento das condições organizacionais que asseguram a oferta de cuidados de saúde seguros. Após cinco etapas de validação, o Questionário AGRASS foi considerado relevante, válido, útil e viável para mensurar a implantação da gestão dos riscos assistenciais em serviços de saúde brasileiros. O questionário pode ser utilizado para: avaliar um serviço de saúde quanto à implantação de práticas de gestão de riscos assistenciais, para guiar o planejamento de intervenções de melhoria; reavaliar um serviço de saúde, para mensurar a melhoria após realizar intervenções de implantação de práticas de gestão de riscos assistenciais; comparar serviços de saúde individuais ou agregados (por ex., região, município ou estado) quanto ao nível de implantação das práticas de gestão de riscos assistenciais. O instrumento pode ser aplicado por avaliadores externos (inspetores sanitários, certificadores, acreditadores e auditores, entre outros) ou em iniciativas de autoavaliação do serviço de saúde.

### Contribuições para a Segurança do Paciente

O Questionário AGRASS atende a uma necessidade internacional e nacional. Os sistemas de informação são uma das seis principais intervenções estratégicas que a OMS estimula para a qualidade do cuidado em sistemas de saúde^9^e, particularmente no Programa de Segurança do Paciente da OMS, a mensuração da segurança é um objetivo prioritário^24^. No entanto, mesmo países bem desenvolvidos em segurança do paciente como a Inglaterra têm sido instados a melhorar a captação de informação sobre segurança do paciente^5^. Essa oportunidade de melhoria também ficou evidente em uma avaliação das decisões governamentais relacionadas à segurança do paciente em países da Organização para a Cooperação e Desenvolvimento Econômico (OCDE), que mostrou que o eixo de avaliação e monitoramento é um dos menos avançados^25^.

No Brasil, embora a avaliação e monitoramento do PNSP tenham sido previstas^26^, existe ampla margem para melhoria neste aspecto. O Sistema de Notificações para a Vigilância Sanitária (Notivisa) e a avaliação anual de práticas de segurança do paciente implantados pela Anvisa são importantes, porém insuficientes^26 , 27^. Assim, o Questionário AGRASS apresenta-se como uma alternativa de instrumento para a produção de informações para a tomada de decisão em segurança do paciente baseada em 40 itens, que podem ser comparáveis a 38 indicadores simples e 2 indicadores compostos (itens 15 e 30). Esse novo instrumento pode ser utilizado para que os profissionais mensurem a segurança do paciente no nível em que ela apresenta maior carência de aferição, que é o seu componente organizacional. A qualidade do cuidado deve ser monitorada a partir de uma perspectiva sistêmica, incluindo o nível técnico do cuidado individual, mas também os níveis organizacionais do estabelecimento de saúde e do sistema de saúde^18^. Indicadores de segurança do paciente têm sido propostos para todos os níveis^28 , 29^, porém poucos são baseados em critérios organizacionais. A mensuração da segurança deve ser integral, não apenas baseada em resultados ou eventos adversos^28 , 30^. Mensurar a qualidade do cuidado não é fácil e exige equilíbrio.

### A Validade do Questionário AGRASS

As etapas de validação por especialistas possibilitaram alcançar os objetivos de construção e validação do conteúdo do Questionário AGRASS. Além da extensa revisão da literatura, os itens finais foram aprovados quanto à validade de face, que é a importância lógica e aparente para a gestão de riscos, a validade de conteúdo, que é a capacidade de cada item medir o conceito expresso de cada dimensão e subdimensão do questionário, e a validade de construto, que mede o quanto os itens se ajustam à estrutura dimensional do questionário. A validação de conteúdo busca valorizar os formadores de opinião e reconhece a importante contribuição dos *experts* que conhecem o tema^23^. Sobre a confiabilidade, embora tenha tido consistência interna aceitável, ressalta-se que depende do treinamento do avaliador e da correta aplicação do instrumento, exigindo e avaliando criteriosamente os comprovantes necessários. Além disso, os valores da confiabilidade composta reforçam este atributo.

O nome Questionário AGRASS enfatiza a gestão de riscos assistenciais, porém ele deve ser compreendido como um instrumento de avaliação da gestão da segurança do paciente baseado em modelos de gestão de riscos e da qualidade. Quanto à dimensão da estrutura, destaca-se a estruturação de sistemas de liderança para a segurança do paciente e de um ambiente caracterizado por uma cultura organizacional favorável. Sobre a dimensão referente aos processos de gestão de riscos, os que foram incluídos (identificação, análise e avaliação, tratamento e comunicação) aparecem direta ou indiretamente na maioria dos modelos de gestão de riscos e gestão da qualidade^13 , 16 , 28^. A subdimensão de identificação de riscos inclui métodos retrospectivos (como indicadores), em tempo real (como *checklists* ) e prospectivos (como a ronda de segurança)^3 , 13^. Outra questão enfatizada pelo modelo é que os processos não devem ser independentes, mas integrados entre si, algo enfatizado na subdimensão “integração dos processos”.

O fato de o Questionário AGRASS ter sido originalmente desenvolvido para serviços de saúde brasileiros favorece sua validade no contexto local. Outros instrumentos com objetivos parecidos foram desenvolvidos no exterior para a avaliação da gestão de riscos clínicos^15^e da gestão da qualidade^14^, porém não foram adaptados transculturalmente para o Brasil. Embora esses instrumentos pareçam a princípio semelhantes ao AGRASS, têm importantes diferenças no seu modelo conceitual e itens.

### O Sistema AGRASS de Avaliação Eletrônica

Visando facilitar a utilização periódica do Questionário AGRASS, o projeto buscou combinar inovação tecnológica para superar barreiras frequentes à institucionalização de processos de avaliação de serviços de saúde no Brasil. Como nem sempre os serviços de saúde dispõem de pessoas com tempo e habilidades para realizar coleta, análise e produção de relatórios para guiar a tomada de decisão, o Sistema AGRASS facilita esses processos mediante a disponibilização de um aplicativo *mobile* em sistemas Android e iOS. O aplicativo proporciona automaticamente as tabelas de caracterização do serviço e de conformidade em relação aos itens, dimensões e subdimensões, além de gráficos de auxílio à tomada de decisão. O módulo *web* permite agregar avaliações individuais para mensurar a implantação em uma rede de serviços ou região geográfica. Os relatórios são gerados em arquivos .pdf e os bancos de dados em arquivo .xls, sendo estes últimos disponibilizados para análises mais aprofundadas.

### Limitações e Estudos Futuros

O Questionário AGRASS não pretende descrever exaustivamente todas as práticas de gestão da segurança, mas aquelas mais recomendadas internacionalmente e essenciais nacionalmente. Embora possa ser utilizado para auxiliar a inspeção de normas sanitárias, o instrumento não é um roteiro completo de inspeção, pois se limita a guiar a avaliação da gestão de riscos assistenciais.

Sugere-se a realização futura de estudos descritivos da aplicação deste instrumento em diferentes regiões do contexto nacional, além de análises de fatores associados aos bons ou maus resultados de implantação da gestão de riscos assistenciais.

## CONCLUSÃO

O Questionário AGRASS é um instrumento considerado válido após cinco etapas de validação e potencialmente útil para a vigilância e mensuração da estrutura organizacional para a segurança do paciente em serviços de saúde brasileiros. O Sistema AGRASS, composto por um módulo *mobile* e outro módulo *web* , permite uma análise eficiente da implantação da gestão de riscos assistenciais, facilitando a institucionalização da avaliação com foco na segurança do paciente em serviços e sistemas de saúde, que é um componente prioritário e indispensável para a qualidade do cuidado de saúde.
